# High‐grade B‐cell lymphoma developed during the treatment of chronic myeloid leukemia with bosutinib

**DOI:** 10.1002/ccr3.3770

**Published:** 2021-01-13

**Authors:** Teruhito Takakuwa, Ryota Sakai, Shiro Koh, Hiroshi Okamura, Satoru Nanno, Yasuhiro Nakashima, Takahiko Nakane, Hideo Koh, Masayuki Hino, Hirohisa Nakamae

**Affiliations:** ^1^ Department of Hematology Graduate School of Medicine Osaka City University Osaka Japan

**Keywords:** bosutinib, chronic myeloid leukemia, high‐grade B‐cell lymphoma, second malignancy, tyrosine kinase inhibitor

## Abstract

Tyrosine kinase inhibitor (TKI) can help to increase the survival time in chronic myeloid leukemia (CML) patients; however, the risk of secondary malignancies due to TKIs is a growing concern. Only few reports showed clinical course of patients who developed lymphoma during TKI therapies. Herein, we report a case of high‐grade B‐cell lymphoma diagnosed in the course of CML treatment with bosutinib. The 75‐year‐old male patient had been diagnosed with CML 25 years ago. After receiving TKIs (imatinib, nilotinib, and bosutinib), he achieved a major molecular response. Over 3 years after starting bosutinib, he was diagnosed with a high‐grade B‐cell lymphoma. A total of six courses of DA‐EPOCH‐R therapy brought complete remission of the lymphoma. Moreover, BCR‐ABL1 transcript copies remained undetectable by RT‐PCR, 8 months after stopping bosutinib. The risk of secondary malignancy due to TKI has been controversial. It is reported that TKI induces irreversible chromosomal abnormalities or chromosome aberrations and inhibits the proliferation or function of T cells, B cells, and NK cells. These mechanisms of TKI may contribute to the development of secondary malignancy. There remains no consensus on the management of secondary lymphoma during TKI therapies. At present, the only alternative is to observe patients receiving TKI treatment cautiously and to treat secondary lymphoma in the same manner as de novo lymphoma.

## Graphical abstract

There remains no consensus on the management of secondary lymphoma during TKI therapies. The only alternative is to observe patients receiving TKI treatment cautiously and to treat secondary lymphoma in the same manner as de novo lymphoma.

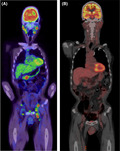

## 1 INTRODUCTION

Chronic myeloid leukemia (CML) accounts for approximately 15‐20% of all leukemias in adults.^1^ CML is characterized by the BCR‐ABL1 fusion gene encoding a constitutively active tyrosine kinase.^2^ Tyrosine kinase inhibitor (TKI) can help to improved the survival time in CML patients to expect almost normal life expectancy; however, the risk of secondary malignancies due to TKIs has not been completely eliminated. It is reported that second malignancies developed in 3.1‐4.5% of case during the treatment course of CML, of which secondary lymphoma accounts for about 5%.^3^, ^4^, ^5^ However, few reports showed clinical course of patients who developed lymphoma during TKI therapies. Herein, we report a case of high‐grade B‐cell lymphoma (HGBCL) diagnosed in the course of CML treatment with bosutinib and present the review of literature.

## 2 CASE

The 75‐year‐old male patient was diagnosed with CML 25 years ago (in August 1994), and he started a treatment with interferon. Twelve years later, the patient was started on imatinib. In October 2009, he gradually developed cytopenia. Although there were approximately 3% blasts in the bone marrow, cytogenetic analysis revealed double Ph clones. Therefore, the patient was diagnosed with an accelerated phase of CML and the treatment was switched to nilotinib. A cytogenetic response was achieved 3 months after starting treatment with nilotinib, and a major molecular response (MMR) was achieved 2 years after starting nilotinib dosing. The patient developed erythema on the extremities and trunk from the start of nilotinib dosing, and antihistamines were continuously administered; however, because the eruptions became uncontrollable, the treatment was changed to bosutinib (400‐500 mg once daily) in March 2016. The MMR was maintained even after switching to bosutinib.

**FIGURE 1:**
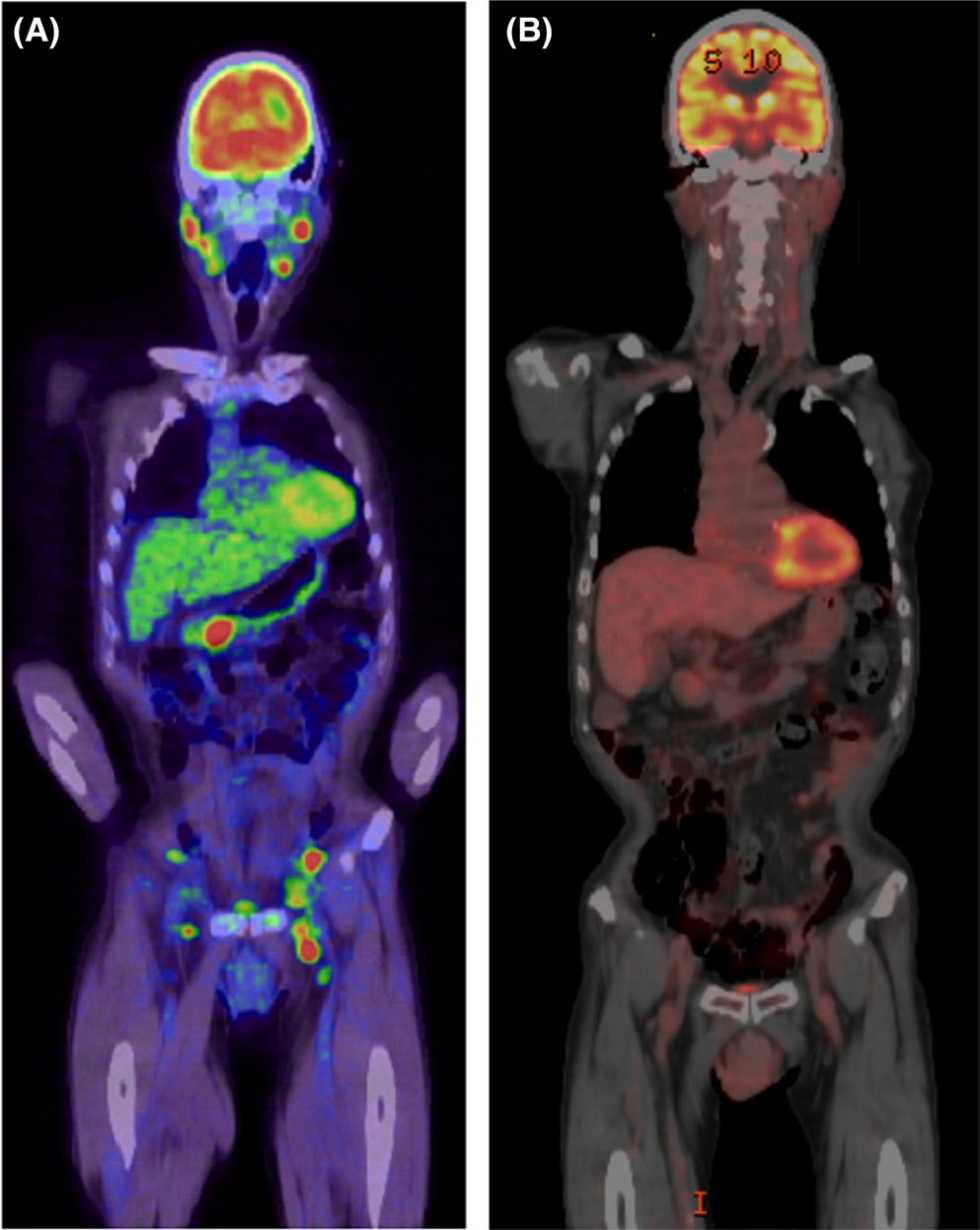
PET/CT images during the diagnosis of the high‐grade B‐cell lymphoma. A) Fused positron emission tomography showing increased fluorodeoxyglucose uptake at the bilateral cervical and also around the pancreas head and bilateral inguinal regions. B) The metabolic CR was maintained after 6 courses of EPOCH‐R therapy

**FIGURE 2:**
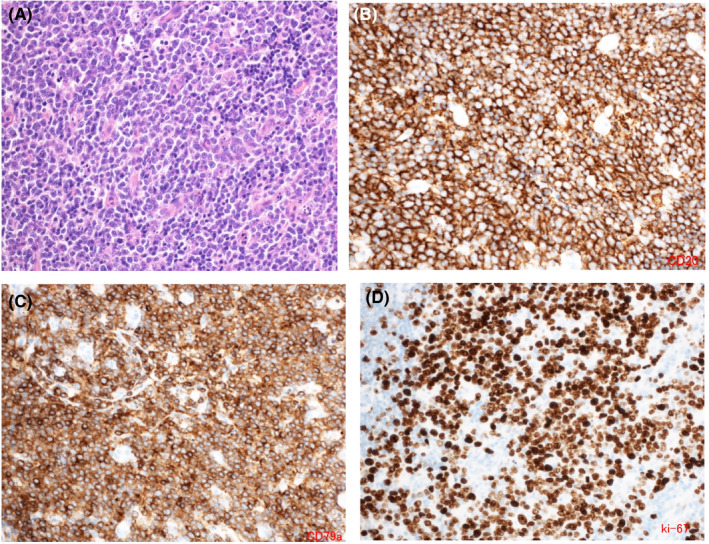
Pathological findings of the inguinal lymph node. Hematoxylin and Eosin staining illustrates cells with large nuclei, proliferating in a starry sky pattern (A). Immunohistochemical staining showed positive for CD20 (B), CD79a (C), and Ki‐67 (D)

In early August 2019, the patient developed a posterior neck pain and malaise and was seen at a local medical institution. Computed tomography (CT) revealed lymphadenopathies in the bilateral cervical, mediastinal, and gastric cardial regions, and also around the pancreas head and bilateral inguinal regions. Positron emission tomography showed abnormal accumulation of fluorodeoxyglucose at these same sites (Figure 1A). Pathological examinations of the inguinal lymph node biopsies showed cells with large nuclei, proliferating in a starry sky pattern, and immunostaining revealed CD19(+), CD20(+), CD79a(+), MUM1(+), BCL‐2(+), c‐myc(+), strongly positive Ki‐67, CD10(−), and TdT(−) (Figure 2A‐D). Neither BCL2 nor MYC rearrangement was detected by fluorescence in situ hybridization. The lymphoma cells were negative for Epstein‐Barr virus (EBV)‐encoded RNA (EBER), which allowed to exclude EBV‐associated lymphomas. There was no bone marrow infiltration, and the patient was diagnosed with a stage III HGBCL. Administration of bosutinib was discontinued since BCR‐ABL1 transcript copies remained below the level of detection achieved by the real‐time quantitative reverse transcription polymerase chain reaction (RT‐PCR). After two courses of the dose‐adjusted EPOCH‐R (etoposide, prednisone, vincristine, cyclophosphamide, doxorubicin, and rituximab) therapy, a complete remission (CR) was confirmed on CT scan. The CR was also maintained after 4 additional courses (a total of 6 courses) of the same therapy (Figure 1B). Moreover, BCR‐ABL1 transcript copies remained undetectable by RT‐PCR 8 months after bosutinib discontinuation.

## 3 DISCUSSION

The present patient developed lymphoma after being treatment with three different TKIs. The patient developed HGBCL after administration of bosutinib. This is a valuable case because no previous studies have reported the detailed clinical course of such a case.

The risk of secondary cancer due to TKI has been a subject of debate. Novartis and MD Anderson Cancer Center reported TKI does not increase the risk of secondary malignancy,^6^, ^7^ but these data are based on spontaneous reports or include patients with diseases other than CML. Contrarily, there have been several reports that the risk of secondary cancer in CML patients who were administered TKI could increase.^3^, ^8^, ^9^ In a survey of 13,256 CML patients, Sasaki K et al reported a 4.5% incidence of secondary malignancies.^4^ In this study, patients who had a history of cancer at the time of CML diagnosis and those who developed other cancers within one year of the CML diagnosis were excluded from this report. Furthermore, the 10‐year risk of secondary malignancy was stable. These reports suggest that TKI may increase the risk of secondary malignancies, and it is therefore necessary to cautiously observe patients receiving a TKI treatment for the onset of other cancers.

Sasaki K et al reported 31 (0.2%) CML patients with secondary lymphoma, but their clinical courses are unknown. Table 1 showed the detailed clinical course of lymphomas that developed during TKI treatment in CML patients.^10^, ^11^, ^12^, ^13^, ^14^, ^15^, ^16^, ^17^ Among them, imatinib was the most commonly used medication, while there were no reports of patients who were treated with bosutinib. The time from the initial TKI dose to the onset of the secondary cancer was longest in our case. The outcomes of the lymphoma are unknown for case 4 and 8, while case 3 died 8 months after developing the lymphoma. Other cases responded well to chemotherapy.

**TABLE 1:** Reported cases of lymphoma in patients with CML treated with TKI

Case	Age	Sex	Year	TKI	Interval	Lymphoma histology	Treatment for lymphoma	Prognosis	Author	Reference
1	65	M	2004	Imatinib	10 mo	MCL	CHOP x5	Die	Rodler E, et al	10
2	53	F	2016	Imatinib	84 mo	EMZBCL	anthracycline‐based	CR	Mihaylov G, et al	11
3	66	F	2017	Dasatinib	2 mo	MBCL	NA	NA	Takeyasu Y, et al	12
4	60	M	2018	Imatinib	8 mo	DLBCL	R‐EPOCH x2, R‐CHOP x4	CR	Abuelgasim KA, et al	13
5	50	M	2018	Imatinib	36 mo	FL	Rituximab	CR	Fujiwara SI, et al	14
6	50	M	2019	Imatinib Dasatinib	120 mo	HL	ABVD x6	CR	Gajendra S, et al	15
7	63	M	2019	Imatinib Nilotinib	84 mo	DLBCL	RCOP + Lenalidomide	CR	Cai Z, et al	16
8	8	M	2019	Imatinib Dasatinib	45 mo	PTNFL	NA	NA	Dominguez‐Pinilla N, et al	17
our case	75	M	2020	Imatinib Nilotinib Bosutinib	161 mo	HGBCL	DA‐EPOCH‐R	CR		

Note

TKI; tyrosine kinase inhibitor, M; male, F; female, MCL; mantle cell lymphoma, EMZBCL; extranodal margial zone B‐cell lymphoma, MBCL; mediastinal B‐cell lymphoma, DLBCL; diffuse large B‐cell lymphoma, FL; Follicular lymphoma, HL; Hodgkin lymphoma, PTNFL; Paediatric‐type nodal follicular lymphoma, HGBCL; high‐grade B‐cell lymphoma, NA; not available, CR; complete response.

Several possible mechanisms of the onset of other hematologic malignancies in CML patients under TKI therapy may be thought possible. First, CML itself may increase the risk of cancer onset. It is possible that BCR‐ABL translocation may introduce genetic instability and that progenitors of other hematologic malignancies were already latent at the time of CML diagnosis.^18^ However, patients who had been in long‐term remission have also been observed with secondary cancer; therefore, this hypothesis alone does not fully explain the mechanisms of onset. Second, TKI‐induced immunosuppression may leave patients vulnerable to secondary cancers. TKIs are known to inhibit the proliferation or function of T cells, B cells, and NK cells,^19^ and this may decrease tumor immunity, thereby contributing to the cancer onset. It is well known that long‐term immunosuppression led to increase the risk of lymphoproliferative complications and secondary hematologic malignancies, in which the most common malignancies are non‐Hodgkin lymphomas.^20^, ^21^ Thus, as long as patients are on TKI therapy, they must be considered as being exposed to the risk of secondary malignancies. Third, EBV can contribute to the pathogenesis of lymphoma, particularly in compromised patients^22^; however, Epstein‐Barr encoding region in situ hybridization was negative with this patient's specimens. In the revised 4th edition of the 2016 classification of the World Health Organization,^23^ a positive expression of EBER in pathological samples is necessary for the diagnosis of EBV + DIBCL. Moreover, there seems also to be concern about TKI‐associated follicular lymphoid hyperplasia.^24^ TKIs may promote B‐cell activation and proliferation resulting from activation of the serine‐threonine kinase Akt (also known as protein kinase B), which may induce clonal abnormalities in B cells.^25^

Dasatinib is a dual‐specific SRC and ABL inhibitor and has 100‐300 fold higher activity than imatinib.^26^, ^27^ Bostinib is also a dual SRC/ABL inhibitor and inhibits the ABL T315I mutant almost 70‐fold more potently than dasatinib.^28^ In contrast to dasatinib, bostinib has reduced activity against nonspecific molecular targets such as KIT and PDGFR (two common off‐targets).^29^ Since TKIs have different off‐target effects, the relevant TKI with the presence of lymphoid hyperplasia or lymphoproliferative disorder should be switched to another TKI or discontinued if possible.

There are no clear reports on the prognosis of lymphoma developed during TKI therapy. The present patient responded rapidly to initial therapy as well as most of other patients displayed in Table 1. Lymphoma arising in patients with primary immunodeficiencies is generally known to have poor a prognosis.^30^ However, the prognosis of most of iatrogenic immunodeficiency‐associated lymphomas is not poor.^31^ Presently, the only treatment available for lymphomas resulting from TKI therapy is the same as that for de novo lymphoma. Accumulating data from more patients is needed for the development of novel therapeutic strategies for lymphomas secondary to TKI.

## 4 ETHICS STATEMENT

A written informed consent was obtained from the patient for publication.

## CONFLICT OF INTEREST

The authors have no conflicts of interest to declare for publication of this article.

## AUTHOR CONTRIBUTION

Teruhito Takakuwa: wrote the manuscript with support from Hirohisa Nakamae. Ryota Sakai: designed a figure and a table. All authors: discussed the case and contributed to the final manuscript.

## Data Availability

The data of this case are available from the corresponding author, TT, upon reasonable request.

## References

[1] Siegel RL , Miller KD , Jemal A . Cancer statistics, 2017. CA Cancer J Clin. 2017;67(1):7‐30. 10.3322/caac.21387 28055103

[2] Sattler M , Griffin JD . Mechanisms of transformation by the BCR/ABL oncogene. Int J Hematol. 2001;73(3):278‐291. 10.1007/bf02981952 11345193

[3] Roy L , Guilhot J , Martineau G , Larchee R , Guilhot F . Unexpected occurrence of second malignancies in patients treated with interferon followed by imatinib mesylate for chronic myelogenous leukemia. Leukemia. 2005;19(9):1689‐1692. 10.1038/sj.leu.2403874 16015386

[4] Sasaki K , Kantarjian HM , O'Brien S , et al. Incidence of second malignancies in patients with chronic myeloid leukemia in the era of tyrosine kinase inhibitors. Int J Hematol. 2019;109(5):545‐552. 30830579 10.1007/s12185-019-02620-2 PMC11694142

[5] Krishnan B , Morgan GJ . Non‐Hodgkin lymphoma secondary to cancer chemotherapy. Cancer Epidemiol Biomarkers Prev. 2007;16(3):377‐380. 10.1158/1055-9965.epi-06-1069 17372233

[6] Pilot PR , Sablinska K , Owen S , Hatfield A . Epidemiological analysis of second primary malignancies in more than 9500 patients treated with imatinib. Leukemia. 2006;20(1):148. 16292349 10.1038/sj.leu.2404025

[7] Verma D , Kantarjian H , Strom SS , et al. Malignancies occurring during therapy with tyrosine kinase inhibitors (TKIs) for chronic myeloid leukemia (CML) and other hematologic malignancies. Blood. 2011;118(16):4353‐4358. 10.1182/blood-2011-06-362889 21846902 PMC3291487

[8] Shah BK , Ghimire KB . Second primary malignancies in chronic myeloid leukemia. Indian J Hematol Blood Transfus. 2014;30(4):236‐240. 10.1007/s12288-013-0328-2 25435720 PMC4243416

[9] Gunnarsson N , Stenke L , Hoglund M , et al. Second malignancies following treatment of chronic myeloid leukaemia in the tyrosine kinase inhibitor era. Br J Haematol. 2015;169(5):683‐688. 10.1111/bjh.13346 25817799

[10] Rodler E , Welborn J , Hatcher S , et al. Blastic mantle cell lymphoma developing concurrently in a patient with chronic myelogenous leukemia and a review of the literature. Am J Hematol. 2004;75(4):231‐238. 10.1002/ajh.20025 15054816

[11] Mihaylov G , Varbanova V , Stoeva V , Dikov T . Extranodal marginal zone B‐cell lymphoma arising in chronic myeloid leukaemia successfully treated with tyrosine kinase inhibitor: a case report. Hippokratia. 2016;20(3):241‐243. 29097894 PMC5654445

[12] Takeyasu Y , Satake A , Azuma Y , et al. Tyrosine kinase inhibitor and rituximab‐CHOP treatment for concurrent chronic myeloid leukemia and non‐Hodgkin lymphoma: a case report. Clin Case Rep. 2017;5(12):2047‐2050. 10.1002/ccr3.1253 29225854 PMC5715580

[13] Abuelgasim KA , Rehan H , Alsubaie M , et al. Coexistence of chronic myeloid leukemia and diffuse large B‐cell lymphoma with antecedent chronic lymphocytic leukemia: a case report and review of the literature. J Med Case Rep. 2018;12(1):64. 10.1186/s13256-018-1612-4 29524963 PMC5845776

[14] Fujiwara SI , Shirato Y , Ikeda T , et al. Successful treatment of follicular lymphoma with second‐generation tyrosine kinase inhibitors administered for coexisting chronic myeloid leukemia. Int J Hematol. 2018;107(6):712‐715. 10.1007/s12185-017-2378-y 29185155

[15] Gajendra S , Sharma A , Sharma R , Gupta SK , Sood N , Sachdev R . Hodgkin lymphoma in a case of chronic myeloid leukemia treated with tyrosine kinase inhibitors. Turkish J Pathol. 2016;35(1):74‐78. 10.5146/tjpath.2016.01368 28272671

[16] Cai Z , Liu S , Zi J , Ma J , Ge Z . A case of primary gastric diffuse large B‐cell lymphoma occurring in chronic myeloid leukemia. OncoTargets Ther. 2019;12:5917‐5923. 10.2147/ott.s212838 PMC6660623 31413589

[17] Dominguez‐Pinilla N , Martinez‐Zamorano E , Campos‐Martin Y , et al. Paediatric‐type nodal follicular lymphoma in a child diagnosed with chronic myeloid leukaemia. Br J Haematol. 2019;186(6):e207‐e209. 10.1111/bjh.16089 31286492

[18] Fabarius A , Kalmanti L , Dietz CT , et al. Impact of unbalanced minor route versus major route karyotypes at diagnosis on prognosis of CML. Ann Hematol. 2015;94(12):2015‐2024. 10.1007/s00277-015-2494-9 26385387

[19] Haguet H , Douxfils J , Chatelain C , Graux C , Mullier F , Dogne JM . BCR‐ABL Tyrosine Kinase Inhibitors: Which Mechanism(s) May Explain the Risk of Thrombosis? TH Open. 2018;2(1):e68‐e88. 10.1055/s-0038-1624566 31249931 PMC6524858

[20] Filipovich AH , Mathur A , Kamat D , Kersey JH , Shapiro RS . Lymphoproliferative disorders and other tumors complicating immunodeficiencies. Immunodeficiency. 1994;5(2):91‐112. 8032367

[21] Tran H , Nourse J , Hall S , Green M , Griffiths L , Gandhi MK . Immunodeficiency‐associated lymphomas. Blood Rev. 2008;22(5):261‐281. 10.1016/j.blre.2008.03.009 18456377

[22] Shannon‐Lowe C , Rickinson AB , Bell AI . Epstein–Barr virus‐associated lymphomas. Philos Transact Royal Society B Biol Sci. 2017;372(1732):20160271‐ 10.1098/rstb.2016.0271 PMC5597738 28893938

[23] Swerdlow SH , Campo E , Pileri SA , et al. The 2016 revision of the World Health Organization classification of lymphoid neoplasms. Blood. 2016;127(20):2375‐2390. 10.1182/blood-2016-01-643569 26980727 PMC4874220

[24] Bouquet E , Jourdain A , Machet MC , Beau‐Salinas F , Jonville‐Béra AP . Dasatinib‐associated follicular lymphoid hyperplasia: First pediatric case report and literature review. Pediatric Blood Cancer. 2017;64(11):e26597. 10.1002/pbc.26597 28439970

[25] Li HL , Davis WW , Whiteman EL , Birnbaum MJ , Puré E . The tyrosine kinases Syk and Lyn exert opposing effects on the activation of protein kinase Akt/PKB in B lymphocytes. Proc Natl Acad Sci U S A. 1999;96(12):6890‐6895. 10.1073/pnas.96.12.6890 10359809 PMC22012

[26] Shah NP , Tran C , Lee FY , Chen P , Norris D , Sawyers CL . Overriding imatinib resistance with a novel ABL kinase inhibitor. Science (New York, NY). 2004;305(5682):399‐401. 10.1126/science.1099480 15256671

[27] Tokarski JS , Newitt JA , Chang CY , et al. The structure of Dasatinib (BMS‐354825) bound to activated ABL kinase domain elucidates its inhibitory activity against imatinib‐resistant ABL mutants. Cancer Res. 2006;66(11):5790‐5797. 10.1158/0008-5472.can-05-4187 16740718

[28] Remsing Rix LL , Rix U , Colinge J , et al. Global target profile of the kinase inhibitor bosutinib in primary chronic myeloid leukemia cells. Leukemia. 2009;23(3):477‐485. 10.1038/leu.2008.334 19039322

[29] Puttini M , Coluccia AM , Boschelli F , et al. In vitro and in vivo activity of SKI‐606, a novel Src‐Abl inhibitor, against imatinib‐resistant Bcr‐Abl+ neoplastic cells. Cancer Res. 2006;66(23):11314‐11322. 10.1158/0008-5472.can-06-1199 17114238

[30] Parvaneh N , Filipovich AH , Borkhardt A . Primary immunodeficiencies predisposed to Epstein‐Barr virus‐driven haematological diseases. Br J Haematol. 2013;162(5):573‐586. 10.1111/bjh.12422 23758097

[31] Tokuhira M , Watanabe R , Nemoto T , et al. Clinicopathological analyses in patients with other iatrogenic immunodeficiency‐associated lymphoproliferative diseases and rheumatoid arthritis. Leukemia Lymphoma. 2012;53(4):616‐623. 10.3109/10428194.2011.625101 21933041

